# The Chloride Conductance Inhibitor NS3623 Enhances the Activity of a Non-selective Cation Channel in Hyperpolarizing Conditions

**DOI:** 10.3389/fphys.2021.743094

**Published:** 2021-10-11

**Authors:** David Monedero Alonso, Laurent Pérès, Aline Hatem, Guillaume Bouyer, Stéphane Egée

**Affiliations:** ^1^Sorbonne Université, CNRS, UMR LBI2M, Station Biologique de Roscoff SBR, Roscoff, France; ^2^Laboratory of Excellence GR-Ex, Paris, France

**Keywords:** erythrocyte, membrane potential, red blood cell, CCCP method, chloride channel inhibitor, non-selective cation channel

## Abstract

Handbooks of physiology state that the strategy adopted by red blood cells (RBCs) to preserve cell volume is to maintain membrane permeability for cations at its minimum. However, enhanced cation permeability can be measured and observed in specific physiological and pathophysiological situations such as *in vivo* senescence, storage at low temperature, sickle cell anemia and many other genetic defects affecting transporters, membrane or cytoskeletal proteins. Among cation pathways, cation channels are able to dissipate rapidly the gradients that are built and maintained by the sodium and calcium pumps. These situations are very well-documented but a mechanistic understanding of complex electrophysiological events underlying ion transports is still lacking. In addition, non-selective cation (NSC) channels present in the RBC membrane have proven difficult to molecular identification and functional characterization. For instance, NSC channel activity can be elicited by Low Ionic Strength conditions (LIS): the associated change in membrane potential triggers its opening in a voltage dependent manner. But, whereas this depolarizing media produces a spectacular activation of NSC channel, Gárdos channel-evoked hyperpolarization's have been shown to induce sodium entry through a pathway thought to be conductive and termed *P*_cat_. Using the CCCP method, which allows to follow fast changes in membrane potential, we show here (i) that hyperpolarization elicited by Gárdos channel activation triggers sodium entry through a conductive pathway, (ii) that chloride conductance inhibition unveils such conductive cationic conductance, (iii) that the use of the specific chloride conductance inhibitor NS3623 (a derivative of Neurosearch compound NS1652), at concentrations above what is needed for full anion channel block, potentiates the non-selective cation conductance. These results indicate that a non-selective cation channel is likely activated by the changes in the driving force for cations rather than a voltage dependence mechanism *per se*.

## Introduction

The most prominent feature of erythrocyte ionic permeability is the selectivity for anions. Hydrophilic anions such as Cl^−^ and ${\text{HCO}}_{3}^{-}$ cross the membrane about one million times faster than hydrophilic cations of similar size such as Na^+^ or K^+^. Thus, human erythrocytes display a relatively high chloride conductance (G_Cl_-) of about 25 *μ*S.cm^−2^ (Bennekou, 1999). The dominance of G_Cl_- over the other conductance's clamps the membrane potential of red blood cells (RBCs) close to the Nernst equilibrium for Cl^−^ (−12 mV), facilitating in this way CO_2_ transport within the blood owing to fast hydration of this gas within RBCs by the carbonic anhydrase coupled to the fast exchange of produced bicarbonate through the chloride/bicarbonate exchanger AE1 or Band 3, the so-called “chloride shift” (Hoffman and Geibel, 2005). Studies on chloride channels present in RBCs membrane regained interest when anion channels could potentially be targeted to eradicate malaria (Kirk, 2000). These works done using patch-clamp allowed to revisit the molecular nature of anion channels in RBCs (Egee et al., 2002; Huber et al., 2002). Several candidates have been proposed among them CFTR (Verloo et al., 2004), ClC-2 (Huber et al., 2004; Bouyer et al., 2007), VDAC (Bouyer et al., 2011). However, the exact nature of the chloride channels involved in the membrane conductance is still elusive. Cation movements, on the contrary, must stay low in order to maintain homeostasis and rheological properties constant in the steady state. Small leaks are swiftly corrected for by the action of powerful pumps such as the 3Na^+^/2K^+^ pump and the Plasma Membrane Ca^2+^ ATPase (PMCA), (Tosteson and Hoffman, 1960). However, the human red cell membrane is endowed with numerous cationic permeability's, notably ionic channels, that may represent a threat if their activity and generated fluxes are above pump capacity (Thomas et al., 2011). Among the cationic channels, the human red blood cell membrane contains 150 to 200 copies of a K^+^ channel activated by Ca^2+^ ions (IK1, KCa3.1, KCNN4). The so-called Gárdos channel is the most well-described in the literature (Hamill, 1983; Maher and Kuchel, 2003; Lew and Tiffert, 2017). At intracellular physiological Ca^2+^ concentrations (30 to 60 nM), (Tiffert and Lew, 1997), the Gárdos channel is inactive. The activation threshold is reached at 150 nM and the maximum activity at around 2 *μ*M (Dunn, 1998); the transmembrane K^+^ flux is then multiplied by a thousand: the massive output of K^+^ ions hyperpolarizes the membrane, whose potential then shifts toward the equilibrium potential for K^+^ ions (E_K_) and creates a favorable electrochemical gradient for anion release, leading to fast and massive dehydration.

On the contrary, the picture for Non-Selective Cation channels (NSC) also present in the red cell membrane is rather more complex and their molecular identity remains elusive. Indeed, NSC channels carry Na^+^, K^+^ and Ca^2+^ down their respective electrochemical gradients and may, once active, dissipate the gradient maintained by the pumps. Numerous reports describe these conductance's functionally but it is still unknown whether different types of channels with specific roles exist or, rather, a single NSC can operate under different modes of action. Nevertheless, there is evidence for several channel proteins that may account for the cation exchange observed in physiological or pathophysiological conditions (Bennekou, 1999).

Piezo1, a mechanosensitive pore-forming NSC channel has been shown to be critical for RBC ion homeostasis as its impairment impacts cell hydration (Cahalan et al., 2015) and was reported as the primary link to Hereditary Xerocytosis (HX, OMIM #194380) when mutated (Zarychanski et al., 2012; Bae et al., 2013; Andolfo et al., 2018). Moreover, it has been hypothesized to play a role in capillary passage in association with the Gárdos channel (Dyrda et al., 2010; Faucherre et al., 2014; Danielczok et al., 2017b). The Transient Receptor Potential Channel 6 (TRPC6), a NSC channel member of the TRP superfamily, was detected at the RNA level in erythroid progenitors and at the protein level in mature RBCs in humans and mice (Foller et al., 2008; Danielczok et al., 2017a). N-methyl-D-aspartate Receptors (NMDAR) are ligand-gated NSC channels that have been found in greater numbers in Sickle Cell Disease (SCD) erythrocytes compared to controls (Hänggi et al., 2014). More recently, TRPV2 channels have been elegantly detected in RBC's membrane and proposed as a key element in the maintenance of cellular homeostasis (Belkacemi et al., 2021).

In addition, cation leaks through NSC channels are involved and described in various situations. The non-selective cation conductance observed in sickle cells, termed P_sickle_ (Lew and Bookchin, 2005) mediates calcium influx leading to Gárdos channel activation, which entails massive K^+^ efflux, and to cell dehydration. The increased cation permeability of terminal senescent RBCs, referred to as *P*_cat_, leads to cation gradient reversal with Na^+^ influx exceeding K^+^ influx (Lew et al., 2007; Cueff et al., 2010). The Non-Selective Voltage Dependent Cation channel (NSVDC) has been extensively characterized at the functional level and is largely responsible for the repolarization of RBCs observed when erythrocyte membrane potential is changed to positive values, as it happens when they are immersed in Low Ionic Strength solutions (Bennekou et al., 2004; Moersdorf et al., 2013).

However, in any case, study of the cationic conductance's of RBCs is hindered by the strong anionic conductance (G_Cl_-), so that chloride pathways inhibitors are routinely employed in order to study cation movements. These include, among others, DIDS (4,4′-Diisothiocyano-2,2′-stilbenedisulfonic acid) shown to bind covalently Band 3 and known to inhibit G_Cl_- (Cabantchik and Greger, 1992), NPPB [5-nitro-2-(3-phenylpropylamino)benzoic acid], (Cabantchik and Greger, 1992) and Neurosearch-developed NS1652 [(2-(N8-trifluoromethylphenyl)ureido)-benzoic acid], (Bennekou et al., 2000) and NS3623 [N-[4-bromo-2-(1H-tetrazol-5-yl)phenyl]-N′-(3-trifluoromethyl-phenyl)urea] compounds (Bennekou et al., 2001). The latter was proven, in a seminal work on SCD, to be the most effective chloride inhibitor to date, even more than NS1652, with an IC_50_ of 210 nM vs. 620 nM for NS1652 (Bennekou et al., 2001).

Measuring membrane potential changes via the CCCP (carbonyl cyanide-m-chloro-phenyl-hydrazone) method has proven to be a valuable tool in the study of NSC activity (Macey et al., 1978; Bennekou et al., 2006; Filser et al., 2021; Peres et al., 2021). For instance, inhibition of G_Cl_- in Low Ionic Strength conditions (LIS) revealed NSC channel activity (Bennekou et al., 2003) which are the correlate of NSVDC channels previously described by patch-clamp at the single level (Christophersen and Bennekou, 1991; Kaestner et al., 1999, 2000).

Thus, inhibiting G_Cl_- allows the magnification of the NSC contribution to any changes in membrane potential. Nevertheless, the concentration dependence of the efficiency of these inhibitors is often poorly described. Herein we present data showing that NS3623, the most efficient chloride channel inhibitor, surprisingly acts as an enhancer of the cation conductance at concentrations above 10 *μ*M. Interestingly, this activity also occurs after Gárdos channel-induced hyperpolarization, in conditions where the driving force favors Na^+^ entry at a membrane potential for which NSCs were thought to be in the closed state.

## Materials and Methods

### Reagents

All salts were acquired from Sigma-Aldrich and of analytical grade or better. Nominal calcium-free solutions amount to 4 *μ*M Ca^2+^ due to typical salt contamination (Baunbaek and Bennekou, 2008).

### Solutions

Normal Ringer: 154 mM NaCl, 2 mM KCl. Calcium Ringer: 154 mM NaCl, 2 mM KCl, 1 mM CaCl_2_. Choline-substituted Ringers: **0%**: 154 mM NaCl, 2 mM KCl**. 25%:** 115.5 mM NaCl, 38.5 mM Choline Chloride, 2 mM KCl **50%**: 77 mM NaCl, 77 mM Choline Chloride. 2 mM KCl **100%** 154 mM Choline Chloride, 2 mM KCl.

### Red Blood Cells

Blood from healthy human donors was drawn into heparinized vacuum tubes, washed thrice with unbuffered saline by centrifugation for 5 min at 5,200 rcf, the buffy coat and plasma removed then with a final step of 1 min at 12,000 rcf, and the packed cells stored at 4°C until use.

### Drugs

A23187 [calcymicin; 5-(methylamino)-2-{[(2S, 3R, 5R, 6S, 8R, 9R)-3,5,9-trimethyl-2-[(2S)-1-oxo-1-(1H-pyrrol-2-yl)propan-2-yl]-1,7-dioxaspiro[5.5]undecan-8-yl]methyl]-1,3-benzoxazole-4-carboxylic acid]; Valinomycin [Cyclo(L-Val-D-HyIva-D-Val-L-Lac-)3: HyIva = –Hydroxyisovaleric acid, Lac = Lactic acid]; CCCP (carbonyl cyanide 3-chlorophenylhydrazone), DIDS (4,4′-Diisothiocyano-2,2′-stilbenedisulfonic acid) were purchased from Sigma-Aldrich (Saint Quentin Fallavier, France); NPPB [5-nitro-2-(3-phenylpropylamino)benzoic acid]; NS3623 [N-[4-bromo-2-(1H-tetrazol-5-yl)phenyl]-N′-(3-trifluoromethyl-phenyl)urea] and NS309 (6,7-Dichloro-1H-indole-2,3-dione 3-oxime) were purchased from Tocris (France).

### Membrane Potential Estimation

The CCCP method (Macey et al., 1978; Bennekou and Christophersen, 1986; Peres et al., 2021) or MBE method as renamed recently (Jansen et al., 2021) was used for the monitoring of membrane potential evolution. When erythrocytes are suspended in nominally buffer-free solution in the presence of the protonophore CCCP, changes in extracellular pH reflect membrane potential changes, since protons are kept at equilibrium across the membrane. The membrane potential can thus be estimated from:


$$\begin{array}{l} V_{M}=61.51\;mV\cdot \left(pH_{i}-pH_{o}\right) \end{array}$$

Due to the high red cell buffer capacity, the intracellular pH remains constant (at about 7.2) throughout an experiment and can be estimated as the pH of the solution after lysis with Triton-X-100 at the end of the experiment.

### Experimental Procedure

2900 *μ*l of experimental solution containing 20 *μ*M of CCCP is thermostated at 37°C Under constant magnetic stirring. For each experiment 100 *μ*l of packed red blood cells (99% hematocrit) are added, to reach final cytocrit of 3.3%. All inorganic compounds are added as stock solution 1,000X in DMSO (Dimethyl Sulfoxide). The final concentration of DMSO never exceeds 0.3%, a concentration that has no effect on either fluxes or membrane potential.

Extracellular pH is measured using a G200 pH electrode (Radiometer) coupled to a Red Rod 200 reference electrode (Radiometer) and a PHM210 pHmeter (Radiometer). Sampling and acquisition are done with an electrode amplifier (EA-BTA, Vernier, USA) at a rate of 1 measurement per second connected to an AD LABQUEST Mini interface (Vernier, USA) with a resolution of 0.01 pH unit. The data are visualized and analyzed with the Logger Lite software (Vernier, France).

At the end of each experiment the detergent Triton X-100(1% in 3M NaCl) is added, causing total cell hemolysis and the result is a solution which attains the intracellular pH.

### Relative Chloride Conductance Units

The chloride conductance was calculated based on maximum hyperpolarization's reached after valinomycin treatment [not shown, see Bennekou et al. for details (Bennekou et al., 2000)]. This allows to examine the efficacy of NS3623. Valinomycin is a K^+^ ionophore, hence, the K^+^ conductance increase is constant for a given concentration of valinomycin and since the Cl^−^ ground leak is also constant, the membrane potential reached reflects the activity of both conductive pathways. Accordingly, the anion conductance change can be calculated with known Nernst potentials for K^+^ (*E*_*K*_ = −110*mV*) and Cl^−^ (*E*_*Cl*_ = −12*mV*) and the membrane potential (*V*_*M*_) measured in the presence of valinomycin.


$$\begin{array}{l} G_{K}^{val}=G_{Cl^{0}}\frac{E_{Cl}-V_{M}}{V_{M}-E_{K}} \end{array}$$

$$\begin{array}{l} G_{Cl^{-}}=G_{K}^{val}\frac{V_{M}-E_{K}}{E_{Cl}-V_{M}} \end{array}$$

### Cell Water, Na^+^ and K^+^ Content Determination

0.5 ml aliquots of the cell suspension (10% hematocrit), with drugs (if any) added at indicated times, were distributed in polyethylene micro test tubes and centrifuged at 20,000 × g for 10 min at 4°C. After centrifugation the packed cell mass was separated from the supernatant by slicing the tube with a razor blade below the top of the red cell column.

### Water Content

After weighting, the packed cells were dried to constant weight for at least 24 h at 90°C and re-weighted. RBC volume depends on the intracellular water content, which is estimated to be about 90 fl for a healthy discocyte. Shape change can be misleading in the estimation of the cell's water content due to the great plasticity of the red cell membrane. These measurements are independent of cell shape.

### Na^+^ and K^+^ Content

The packed cells within the sliced tube were lysed in 1 ml MilliQ water. Proteins were denatured to ease separation by addition of 231.9 *μ*M of perchloric acid. The tubes were spun at 16,900 g for 7.5 min at 4°C and the supernatant passed onto sample tubes and diluted 10 times. The ionic content was measured using a flame photometer (PFP7, Jenway). The amounts of Na^+^ or K^+^ measured are reported as *μ*mol/g dry cell solid.

### Statistical Analysis

Data are shown as mean± SEM, number of replicates are stated in figure legends. Some error bars fall within the symbols and are not visible.

Differences between samples was assessed by using *t*-test. Exact *p*-values are stated within the text and represented on figures according to the following code: ^*^, *p* < 0.05; ^**^, *p* < 0.01; ^***^, *p* < 0.001.

## Results

**Figure 1:**
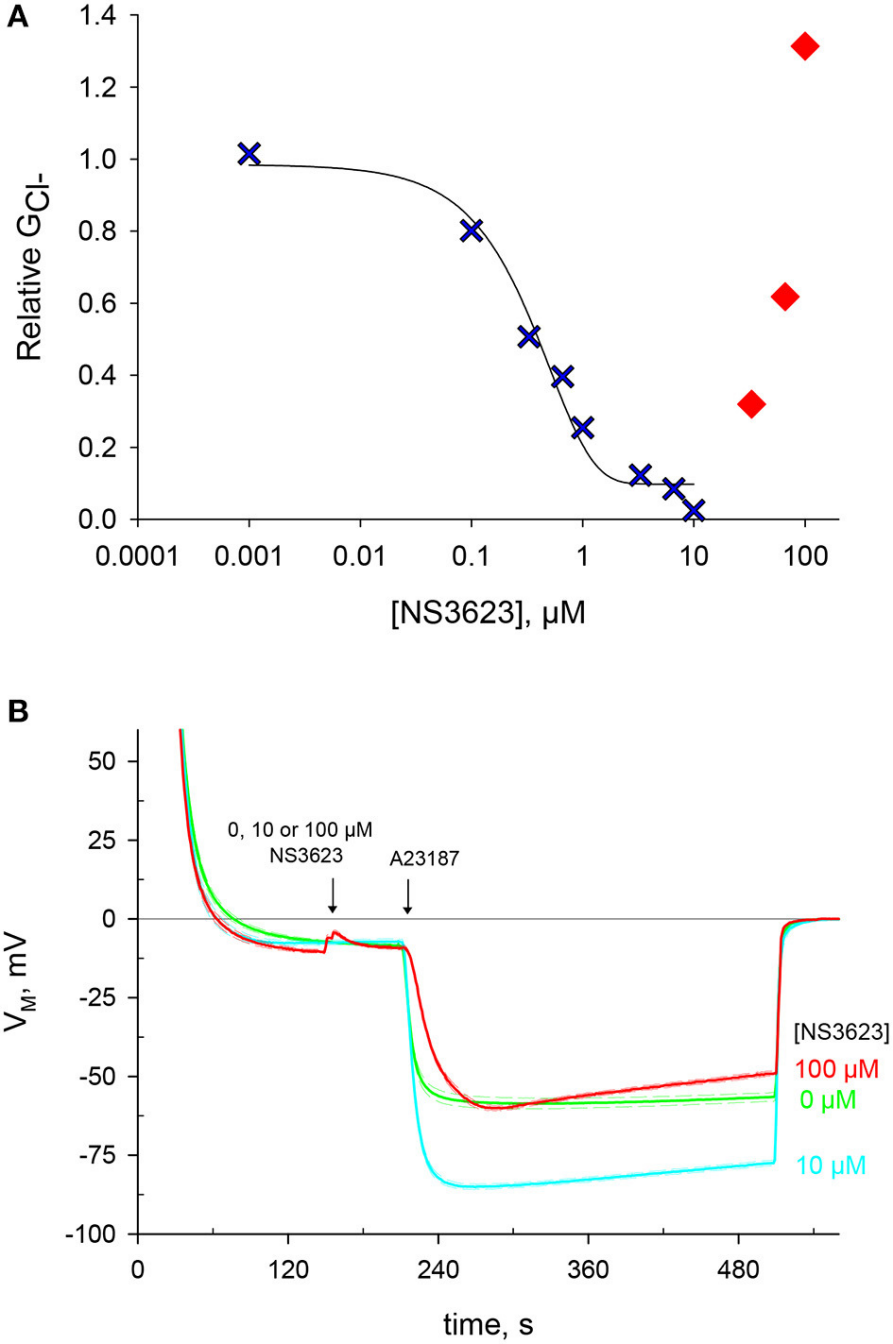
NS3623 inhibits chloride conductance up to 10 *μ*M, another effect is unveiled at higher concentrations. **(A)** Dose-response curve of NS3623 effect on G_Cl−_, measured via valinomycin treatment. In blue, the experimental data can be fitted to a sigmoid curve up to 10 *μ*M. In red, at higher concentrations the points deviate from this curve. **(B)** Evolution of membrane potential from cells injected into normal Ringer solution at *t* = 30 s with exposure to 0 *μ*M (green, mid trace, *n* = 22), 10 *μ*M (cyan, bottom trace, *n* = 12) or 100 *μ*M (red, top trace, *n* = 4) of NS3623 before hyperpolarization was triggered by 10 *μ*M A23187 addition. Dashed lines indicate ± SEM.

NS3623 was previously reported as a chloride conductance inhibitor with a higher affinity than that of the hitherto most effective blocker NS1652 (Bennekou et al., 2001). We set to incorporate this novel compound in our research, as it is the best-known blocker of the human erythrocyte chloride conductance. A range of NS3623 concentrations was tested and the corresponding $G_{Cl^{-}}$ values, determined after valinomycin treatment, were log-transformed, plotted and fitted to a sigmoid equation in a dose-dependent manner (Figure 1A). As reported, the compound is an excellent inhibitor with 99% block of $G_{Cl^{-}}$ at a concentration of 10 *μ*M. However, this inhibition is lost at concentrations above 10 *μ*M. Such deviation suits two different scenarios: either NS3623 loses its affinity for $G_{Cl^{-}}$ targets at concentrations >10 *μ*M, or the reduced maximal hyperpolarization is caused by the activation of a cationic conductive pathway that lets Na^+^ into the cells due to the huge electrochemical gradient brought about by the hyperpolarization. Given the unlikely prospect that a compound with such high affinity would fail to do so at high concentrations that are still within the micro molar range, we set to investigate whether this compound directly enhances the cation conductance.

### Independent Hyperpolarizing Conditions Shed Light on NS3623 High Concentration Effects on Conductive Pathways

Another way to hyperpolarize quickly RBCs is by increasing drastically the Ca^2+^ permeability. This is achieved by employing A23187 (10 *μ*M), a potent calcium ionophore, which will immediately activate the Gárdos channel. Considering that $G_{Cl^{-}}$ and Gárdos channel (if active) are the strongest conductance's of RBCs, the greater the $G_{Cl^{-}}$ inhibition, the more the cell will hyperpolarize as Gárdos channel activity will bring the membrane potential to very negative membrane potential values.

Pretreating cells with 0, 10 or 100 *μ*M NS3623 does not change the resting membrane potential, then once cells are exposed to A23187 a hyperpolarization follows which is dependent on the concentration of NS3623 (Figure 1B and Supplementary Table 1). Whereas, without inhibitor the membrane potential reaches −59.9 ± 1.6 mV (*n* = 22), the use of 10 *μ*M NS3623 brings it to −85.3 ± 0.8 mV (*n* = 12). But again, surprisingly, at 100 *μ*M the hyperpolarization attains only −60.6 ± 1.5 mV (*n* = 4), considerably reduced compared to 10 *μ*M. Strikingly, a delayed onset of the hyperpolarization occurs after A23187 treatment when cells are pre-treated with 100 *μ*M NS3623. The latter observation strengthens the hypothesis of Na^+^ entry being caused by NS3623, considering that valinomycin and A23187 hyperpolarize the cell by two independent mechanisms and that an inhibitor actually increasing the Cl^−^ conductance is not plausible.

### Impermeant Cationic Substitution Confirms the Cationic Nature of Conductive Pathway

**Figure 2:**
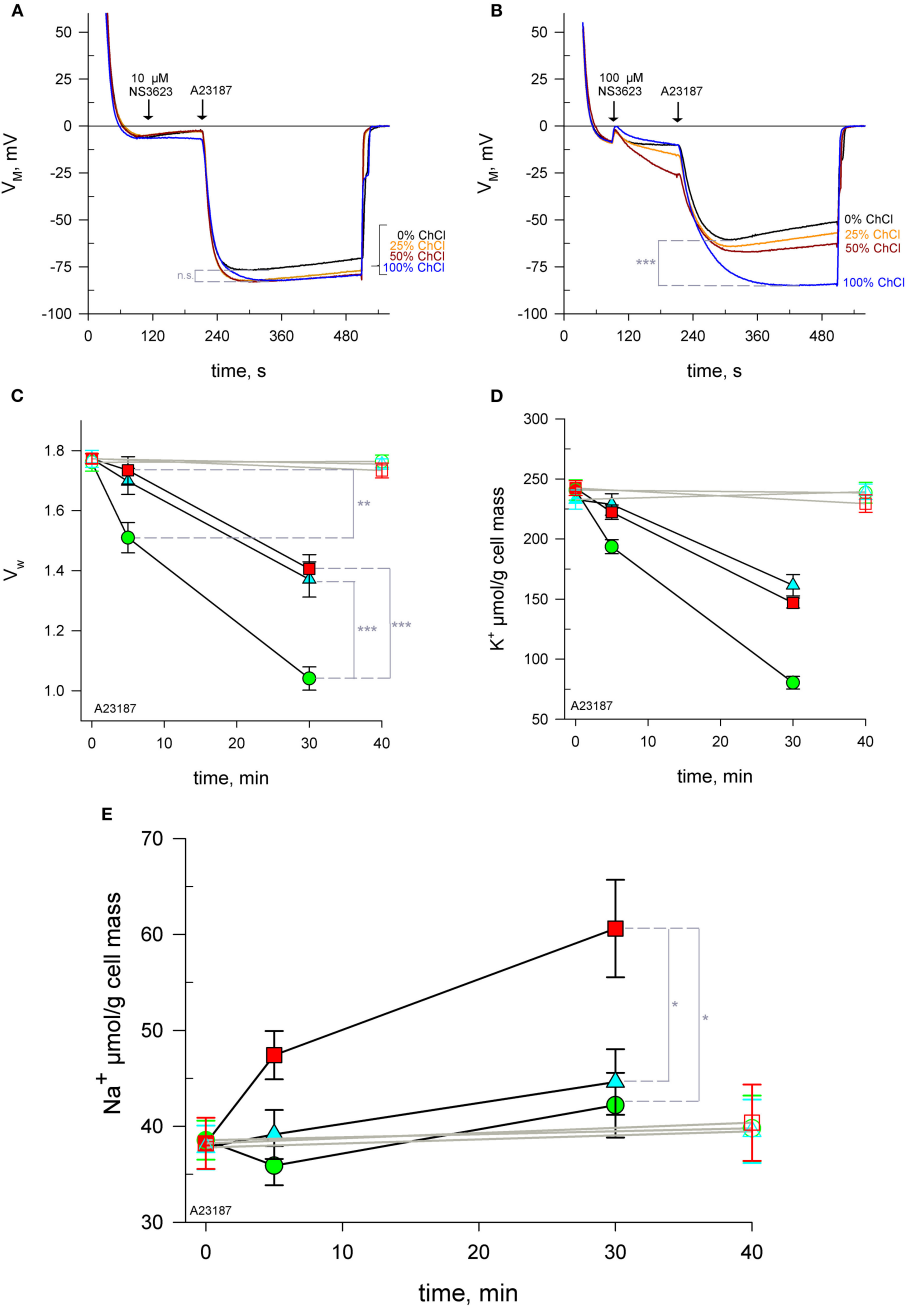
NS3623 becomes a potential NSC activator at high concentrations. **(A, B)** Evolution of membrane potential from cells injected into respective choline chloride-substituted Ringers (0 to 100% choline), treated with 10 *μ*M **(A)**, (*n* = 3) or 100 *μ*M **(B)**, (*n* = 3) of NS3623 and hyperpolarized with A23187 (10 *μ*M). Intracellular water content (wet weight/dry weight), **(C)** and quantity of K^+^ **(D)** and Na^+^ **(E)** in packed RBCs washed with normal Ringer buffered with HEPES (pH = 7.4) and treated with 0 (green), 10 (cyan) or 100 *μ*M (red) of NS3623 and with (closed symbols, black lines) or without (open symbols, gray lines) 10 *μ*M A23187 over time. Bars denote SEM. Average of five independent experiments. n.s., not significant, *p < 0.05, **p < 0.01; ***p < 0.001.

In order to test the hypothesis that Na^+^ enters via a cationic pathway, erythrocytes were suspended in solutions with reduced concentrations of Na^+^, keeping the osmolarity constant by using choline chloride. Choline is a cation unable to cross the plasma membrane through ion channels. Cells were treated with 0, 10 or 100 *μ*M NS3623 followed by A23187. RBCs hyperpolarize to the same extent: −81.7 ± 1.9 mV—regardless of the degree of Na^+^ available when the chloride conductance is inhibited by 10 *μ*M NS3623 (Figure 2A, *n* = 3). At this concentration, no significant difference was found in terms of maximum hyperpolarization between choline-free and choline-containing solutions (*p* = 0.084). However, when RBCs are subjected to 100 *μ*M NS3623, the subsequent hyperpolarization's are dependent on Na^+^ concentration, in an inversely proportional manner: the less Na^+^ present the more the cells hyperpolarize, with the strongest hyperpolarization happening in solutions containing only choline and no Na^+^ (Figure 2B, *n* = 3). Those in 100% Na^+^/0% choline reach (mean ± SEM) −60.8 ± 4.0 mV (*n* = 3) compared to those in 0% Na^+^/100% choline hyperpolarizing to −86.1 ± 0.8 mV (*n* = 3), (*p* < 0.001). In addition, the repolarization observed when Na^+^ is available, is completely absent in solutions devoid of Na^+^ (Figure 2B). Therefore, Na^+^ is involved in the differential effect observed between 10 and 100 *μ*M NS3623. The latter is thought to induce Na^+^ influx via a NSC.

### 100 *μ*M NS3623 Triggers Immediate Na^+^ Influx

To directly confirm this influx, RBCs were treated with 0, 10 or 100 *μ*M NS3623 followed by A23187 and sampled at 5 and 30 mins for subsequent analysis of intracellular water, K^+^ and Na^+^ content. Cells begin to dehydrate immediately after A23187 addition, however the inhibition of the chloride conductance by NS3623 hinders the exit of water as Cl^−^ movement is blocked and there is less osmotically obliged water following KCl exit. After 5 mins, uninhibited cells have lost 14.5% ± 4.4% (*n* = 5) of their internal water compared to 2.2% ± 4.1% (*n* = 5) of 100 *μ*M NS3623 pre-treated cells (*p* = 0.008). Cells untreated with NS3623 dehydrate fast so by 30 mins the dehydration due to Gárdos channel activity is severe, a 41.1% ± 3.6% reduction in water content is observed (Figure 2C) whereas 10 and 100 *μ*M NS3623 pre-treated cells have only lost 22.7% ± 5.8% and 20.8% ± 4.6% respectively (*p* < 0.001).

Dehydration is accounted for by intracellular K^+^ measurements showing a decrease from (mean ± SEM) 240.8 ± 8.5 to 80.4 ± 5.3 *μ*mol/g dry cell mass by 30 mins (*n* = 5, Figure 2D). Both 10 and 100 *μ*M NS3623-treated cells displayed decreased dehydration and K^+^ loss owing to the blocked chloride conductance, as chloride is rate-limiting. This indicates, importantly, that the chloride conductance is indeed inhibited at 100 *μ*M NS3623 as otherwise a greater loss of water and K^+^ would be expected. Na^+^ measurements show that erythrocytes treated with 100 *μ*M NS3623 take in Na^+^ over time after Gárdos channel activation is triggered by A23187 exposure. This Na^+^ influx is increased as early as 5 mins for 100 *μ*M NS3623 pre-treated cells. 30 mins after A23187 treatment, intracellular Na^+^ significantly increased to 60.6 ± 5.2 *μ*mol/g dry cell solid in 100 *μ*M NS3623 pre-treated erythrocytes, whereas 0 and 10 *μ*M NS3623 pre-treated cells contained 42.2 ± 3.4 *μ*mol/g dry cell and 44.6 ± 3.4 *μ*mol/g dry cell solid, respectively (*p* = 0.016 and *p* = 0.031), (Figure 2E). Notably, NS3623 by itself, either at 10 or 100 *μ*M, does not alter water, K^+^ nor Na^+^ content as late as 40 mins after addition, underscoring that hyperpolarizing conditions are a requirement for its enhancing effect on the cation conductance.

### Ca^2+^-Independent Activation of Gárdos Channel Suggests a Ca^2+^ Dependence of NS3623-Activated NSC Conductive Pathway

**Figure 3:**
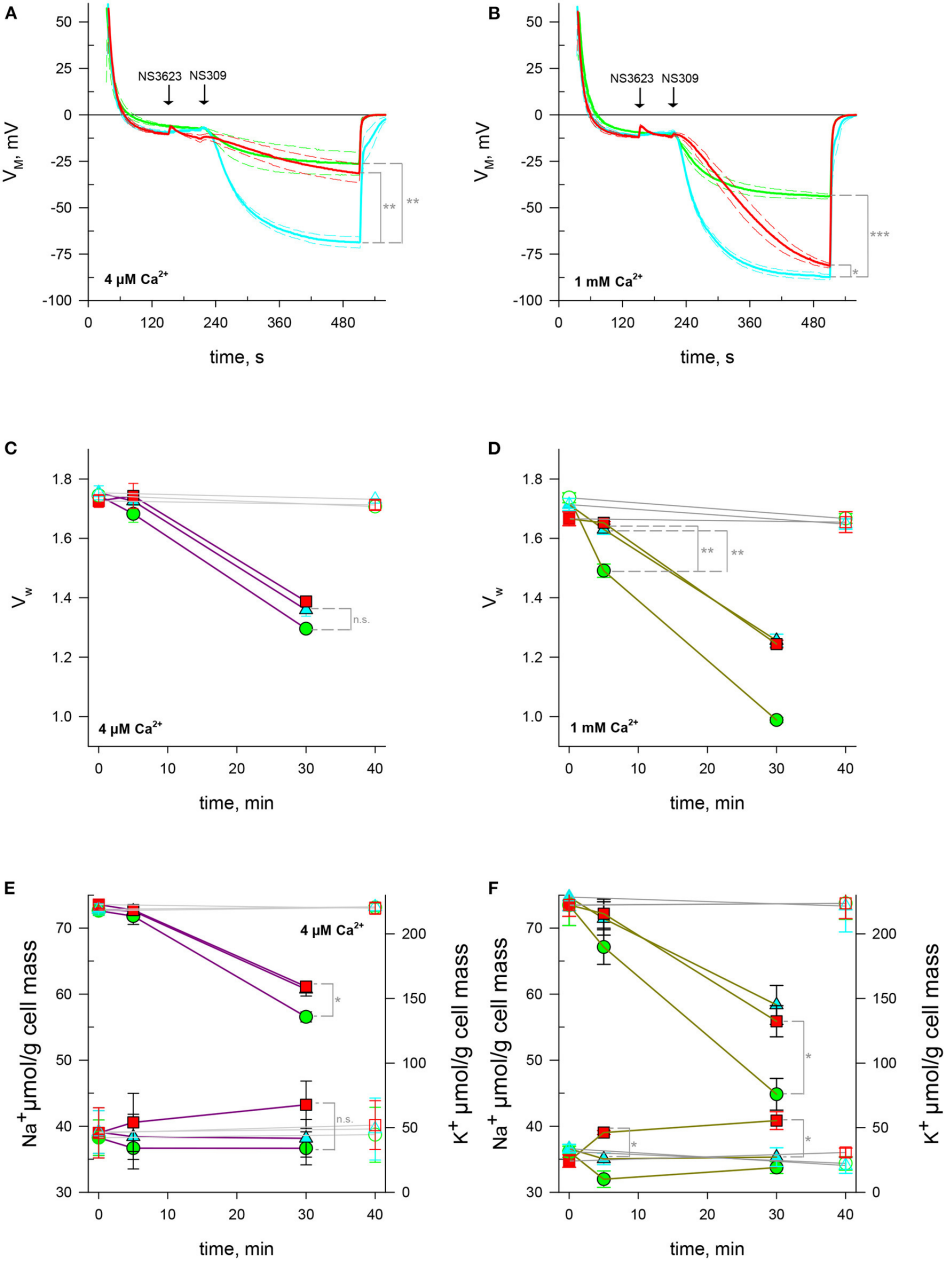
NSC activity enhanced by high concentrations of NS3623 in hyperpolarizing conditions is driving force dependent. **(A, B)** Evolution of membrane potential from cells injected into normal Ringer solutions containing 4 *μ*M **(A)** or 1 mM **(B)** extracellular CaCl_2_, treated with 0 *μ*M (green), 10 *μ*M (cyan) or 100 *μ*M (red) of NS3623 and hyperpolarized with NS309 (100 *μ*M). Average of *n* = 3 experiments, dashed lines indicate ± SEM. **(C–F)** Intracellular water content (wet weight/dry weight), **(C, D)** and quantity of Na^+^ (bottom traces) and K^+^ (top traces), **(E, F)** of red cells treated with 0 (green), 10 (cyan) or 100 *μ*M (red) of NS3623 and with (closed symbols, colored lines) or without (open symbols, gray lines) 100 *μ*M NS309, in the presence of 4 *μ*M **(C, E)** or 1 mM **(D, F)** of extracellular CaCl_2_. Bars denote SEM, average of five **(C, E)** or three **(D, F)** independent experiments. n.s., not significant, *p < 0.05, **p < 0.01; ***p < 0.001.

NS309 is a compound that lowers Gárdos channel sensitivity to calcium so that a smaller intracellular calcium concentration is enough to open the channel. In this manner, NS309 does not increase the intracellular calcium concentration contrary to A23187, allowing for the study of Gárdos channel-induced hyperpolarization's in stable calcium levels (Baunbaek and Bennekou, 2008). To ascertain whether the phenomenon set off by NS3623 is independent on [Ca^2+^]_i_ variation we subjected RBCs to NS309 at two different extracellular [Ca^2+^]_o_: first in conditions meeting those of previous experiments, i.e., 4 *μ*M [Ca^2+^]_o_ and then in a more physiological condition with 1 mM [Ca^2+^]_o_. Our hypothesis postulates that a NSC is activated after treatment with 100 *μ*M NS3623, which entails a Na^+^ increase, as has been shown, and a calcium influx. As expected, hyperpolarization's are much greater when the chloride conductance has been blocked than when it is not: −68.7 ± 3.0 mV (*n* = 3) compared to −26.5 ± 6.3 mV (*n* = 3), (*p* = 0.002). The initial rate of hyperpolarization ($v_{0}^{hyp}$) is also larger: −0.79 ± 0.04 mV/s (*n* = 3) for 10 *μ*M NS3623 pre-treated cells in contrast to −0.21 ± 0.12 mV/s (*n* = 3) of untreated cells (*p* = 0.003). However, when 100 *μ*M NS3623 is used, the hyperpolarization achieved is closer to that of uninhibited cells: −31.5 ± 5.1 mV (*n* = 3) reached by inhibited cells compared to −26.5 ± 6.3 mV (*n* = 3). This is 37.2 mV lower than values reached by 10 *μ*M NS3623-treated cells (*p* = 0.003). 100 *μ*M NS3623 also delays the onset of hyperpolarization with $v_{0}^{hyp}$ −0.09 ± 0.03 mV/s (*n* = 3) in contrast to −0.79 ± 0.04 mV/s (*n* = 3) of RBCs treated with 10 *μ*M NS3623 (*p* < 0.001), (Figure 3A).

The picture changes if these experiments are performed with solutions containing millimolar concentrations of calcium. First, Gárdos channel-induced hyperpolarization becomes maximal, attaining −44.1 ± 1.4 mV (*n* = 3) for 0 *μ*M NS3623-treated cells and −87.3 ± 1.4 for 10 *μ*M treated cells (*p* < 0.001). Even though 100 *μ*M treated cells hyperpolarize to greater extents, −81.2 ± 1.2 mV, getting close to 10 *μ*M NS3623 levels though distinctly different (*p* = 0.028), they do so at a slower pace than 10 *μ*M treated cells: $v_{0}^{hyp}$ is −0.35 ± 0.05 mV/s (*n* = 3) for 100 *μ*M pre-treated RBCs whereas it is −1.29 ± 0.09 mV/s (*n* = 3) for 10 *μ*M pre-treated cells (*p* < 0.001), (Figure 3B). This may be explained by an influx of calcium through the NSC (see discussion).

We quantified intracellular water, Na^+^ and K^+^ after Gárdos channel activation in stable intracellular Ca^2+^ conditions by employing NS309 in low and high extracellular calcium solutions. Contrary to what is observed with A23187, NS3623 has no clear effect on cell volume upon NS309-induced hyperpolarization. Indeed, the dehydration and K^+^ loss are reduced due to Gárdos channel partial activation given the low intracellular concentration dictated by the nominal extracellular calcium concentration (4 *μ*M) and the pumping activity of the PMCA. Cells with $G_{Cl^{-}}$ uninhibited lost 25.7% ± 4.9% (*n* = 5) intracellular water by 30 mins whereas 100 *μ*M NS3623 pre-treated RBCs lost 19.6% ± 4.9% (*n* = 5, *p* = 0.069, Figure 3C). As anticipated, the K^+^ loss at 30 mins was greatest for uninhibited cells with a final content of 128 ± 7.6 *μ*mol/g dry cell mass (*n* = 5) whereas for 100 *μ*M NS3623 pre-treated cells it was 154.5 ± 4.6 *μ*mol/g dry cell mass (*p* = 0.019, *n* = 5, Figure 3E). A small increase in Na^+^, 43.3 ± 6.9 *μ*mol/g dry cell mass is observed once the cells are subjected to 100 *μ*M NS3623, compared to 36.7 ± 2.17 *μ*mol/g dry cell mass for those not treated with NS3623, although it is not significant (*p* = 0.056, Figure 3E, *n* = 5).

Experiments carried out with 1 mM Ca^2+^ Ringers show the high impact of calcium availability on water and cation content when Gárdos channel is opened. As early as 5 mins there is a 14.1% ± 3.0% (*n* = 3) decrease in water content whereas 10 and 100 *μ*M NS3623 pre-treated cells have only lost 4.82% ± 2.76% and 0.80% ± 1.49% respectively (*p* = 0.008 and *p* = 0.003, Figure 3D). This dehydration progresses to extreme levels surpassing those of A23187 by 30 mins for untreated cells. As K^+^ efflux is behind the dehydration, the reduction in K^+^ is equivalent to that of water, with $G_{Cl^{-}}$ moderating K^+^ efflux. By 30 mins, intracellular K^+^ was 75.8 ± 12.3 *μ*mol/g dry cell mass (*n* = 3) for untreated cells in contrast to 132.3 ± 12.0 *μ*mol/g dry cell mass (*n* = 3) in 100 *μ*M NS3623 pre-treated cells (*p* = 0.03, Figure 3F). In these conditions there is a significant Na^+^ increase as soon as 5 mins: cells treated with 10 *μ*M NS3623 have 35.1 ± 0.9 *μ*mol/g dry cell mass (*n* = 3) whereas those treated with 100 *μ*M NS3623 have 39.0 ± 0.3 *μ*mol/g dry cell mass (*p* = 0.013), (*n* = 3). After 30 mins, cells reach 35.3 ± 1.4 *μ*mol/g dry cell mass (*n* = 3) and 40.8 ± 1.4 *μ*mol/g dry cell mass (*n* = 3) for 10 and 100 *μ*M NS3623 treatments, respectively (*p* = 0.012, Figure 3F).

Considering the high accuracy of the measurements, the wide variability observed in experiments conducted with 4 *μ*M Ca^2+^ and the fact that it decreases considerably with 1 mM Ca^2+^ leads us to the tempting conclusion that Ca^2+^ may also influence the NSC *per se*. This assertion is strengthened by the hyperpolarization rate under NS309 and 100 *μ*M NS3623 (see discussion).

Nevertheless, when this is performed with 1 mM extracellular Ca^2+^, the dehydration is fast and extreme (within 30 mins a full dehydration occurs), about 70% of the intracellular K^+^ is lost via Gárdos channel in NS3623-untreated cells whereas only about 40% for NS3623-treated cells. Na^+^ intake increases over time only in 100 *μ*M NS3623-treated cells in a significant manner as early as 5 mins.

## Discussion

Using 100 *μ*M NS3623 prior to hyperpolarization with either valinomycin or A23187 prevents cells from reaching a maximum hyperpolarization, staying at values similar to those untreated with the compound, contrary to the fast hyperpolarization development occurring after 10 *μ*M NS3623 pre-treatment. However, experiments with choline chloride dispelled the possibility that chloride conductance was not inhibited at high concentrations. The Na^+^-dependence points clearly to the emergence of a cation conductance: cells treated with 100 *μ*M NS3623 and 10 *μ*M A23187 in solutions lacking Na^+^ hyperpolarized to similar values as cells treated with 10 *μ*M NS3623 and 10 *μ*M A23187 regardless of the proportions of Na^+^ and choline chloride present.

Moreover, measurements of changes in intracellular water, K^+^ and Na^+^ provide strong evidence on the activation of a non-selective cation conductance by NS3623 at a concentration above 10 *μ*M. It is a matter of fact that a significant Na^+^ influx was reported as measurable after 3 hours when they have been loaded with Ca^2+^ by employing A23187, to activate Gárdos channel and then placed in an ionophore-free solution triggering PMCA-mediated Ca^2+^ extrusion (Lew et al., 2007; Cueff et al., 2010; Lew and Tiffert, 2017). The pathway responsible for such Na^+^ movement, concomitant to cell shrinkage and cell hyperpolarization, was named *P*_cat_ (Lew et al., 2007; Cueff et al., 2010). Interestingly, the reported Na^+^ intake was partially dependent on fast anion exchange possible due to SCN^−^ use whereas we observed very fast Na^+^ intake, in 5 mins, compared to a 3-hours timespan under the influence of a $G_{Cl^{-}}$ inhibitor. In addition, a decrease in Na^+^ uptake is observed upon use of EGTA, hinting at Ca^2+^ involvement in the *P*_cat_ response, even though it was still active in its absence (Tiffert et al., 2007). Considering that under physiological conditions the normal human red cell has a high conductance for chloride, about 25 *μ*S/cm^2^ (Bennekou, 1984; Egee et al., 2002), shrinkage after Gárdos channel activation will be rate-limited by the availability of chloride to accompany the efflux of K^+^. Thus, if the chloride conductance is inhibited, less K^+^ leaves the cell through the Gárdos channel as there is no chloride for electrical compensation and consequently much less water leaves the cell. The fact that 100 *μ*M NS3623-treated cells lose as much water and K^+^ as 10 *μ*M NS3623-treated cells after Gárdos channel activation strengthens the assumption that $G_{Cl^{-}}$ is inhibited at both concentrations. These experiments also showed an increase in intracellular Na^+^ over time only for 100 *μ*M NS3623-treated cells. The only known conductive pathway able to carry large amounts of Na^+^ in such short timespans (as early as 5 mins) in RBCs are Non-Selective Cation Channels (Christophersen and Bennekou, 1991; Kaestner et al., 2000; Duranton et al., 2002).

Importantly, it is worth noting that NS3623 (even at 100 *μ*M) itself does not alter neither membrane potential nor Na^+^ and K^+^ content of RBC at resting membrane potential. NS3623 is still able to inhibit the chloride conductance and to trigger, at high concentrations, NSC activity even if Gárdos channel-elicited hyperpolarization is already fully developed (Supplementary Figure 1). This undoubtedly indicates that NS3623 action relies on the driving force and not on Gárdos channel activation *per se*.

Such behavior indicates either that the effect of the compound is voltage dependent or that the channel enabling this conductance shows voltage dependence. Such an observation suggests that the pathway activated could be the previously described NSVDC (Bennekou et al., 2004). The voltage dependence hypothesis is seemingly ruled out since experiments performed with reduced driving force for K^+^ efflux via Gárdos channel do not alter the repolarization rate when Na^+^ is present (Supplementary Figure 3). This phenomenon remains concentration-dependent at this reduced K^+^ driving force, displaying the same Na^+^ dependence (compare Supplementary Figure 3B with Supplementary Figure 2B).

However, A23187 raises the intracellular Ca^2+^ concentration permanently. In order to decipher whether Ca^2+^ or the driving force triggers NSC activity, we used NS309. This compound increases the sensitivity of Gárdos channel to calcium so that nominal intracellular calcium is sufficient to evoke channel openings (Strobaek et al., 2004) and, more importantly, it keeps the intracellular calcium levels stable and thus maintains the Plasma Membrane Calcium ATPase (PMCA) at resting activity (Baunbaek and Bennekou, 2008). It should be noted that even though the PMCA will keep intracellular calcium levels below the Gárdos channel threshold of activation in physiological conditions, [Ca^2+^]_i_ will indeed be higher than RBCs resuspended in nominal Ca^2+^-free solutions as the PMCA extrusion capacity is greater the more calcium there is, until ATP exhaustion. Thus, NS309 induced-hyperpolarization's are a function of the extracellular calcium concentration (Strobaek et al., 2004; Baunbaek and Bennekou, 2008).

Using NS309 in nominally calcium-free solutions with 0, 10 or 100 *μ*M NS3623 pre-treatment displays smaller overall hyperpolarization's, as the Gárdos channel is not maximally activated, with 0 and 100 *μ*M NS3623-pre-treated cells attaining similar membrane potential values albeit at a slightly faster rate for 100 *μ*M NS3623-pre-treated cells. Nevertheless, performing these experiments with physiological (1 mM) calcium content shows that, amid the greater hyperpolarization's in the context of greater Gárdos channel activation, 100 *μ*M NS3623-pre-treated cells reach values close to those of 10 *μ*M NS3623 cells by 5 mins with the hyperpolarization rate increasing over time. A possible explanation may reside in the fact that the Gárdos channel is not fully activated even with an extracellular calcium concentration of 1 mM and the chloride conductance blocked so when 100 *μ*M NS3623 enhances the activity of the NSC, there is calcium intake, as there is a huge driving force toward the cytoplasm, and Gárdos channel activity increases slightly over time toward the maximum, only in 100 *μ*M NS3623-pre-treated cells. The maximum hyperpolarization difference between low or high calcium solutions was 17.60 ± 3.92 mV (*n* = 3) and 18.65 ± 2.53 mV (*n* = 3) for 0 or 10 *μ*M NS3623-pre-treated cells, whereas the difference for 100 *μ*M NS3623-pre-treated cells reached 49.67 ± 4.21 mV (*n* = 3), highlighting the strong synergistic effect of calcium and NS3623 (100 *μ*M). Such an interplay between Ca^2+^, Gárdos channel and NSVDC was already reported in depolarizing conditions (LIS conditions), (Bennekou et al., 2003).

When calcium is scarce, Gárdos channel activity is limited and water and K^+^ loss is contained. Even though Na^+^ flows in over time in 100 *μ*M NS3623-pre-treated cells, the increase is minimal. However, interestingly, in high calcium solutions the loss of water and K^+^ after NS309 treatment is even more pronounced than for A23187-treated cells and Na^+^ is significantly increased as early as 5 mins only in 100 *μ*M NS3623 pre-treated cells, even though the maximum amounts reached are smaller than those of A23187-treated cells. The amount of intracellular Na^+^ increases after 100 *μ*M NS3623 addition, significantly when cells are resuspended in solutions containing millimolar concentrations of calcium. Thus, there is a NSC-mediated Na^+^ current upon 100 *μ*M NS3623 treatment which electrically counteracts the hyperpolarizing K^+^ loss. Positive charges are leaving (K^+^) and entering (Na^+^) the cell, bringing some electro neutrality even though the Gárdos channel is more powerful which may explain the more pronounced slope as there is a long-term tendency toward E_K+_. This prevents 100 *μ*M pre-treated cells to attain such negative membrane potentials as those of 10 *μ*M NS3623 pre-treated-cells. In nominal Ca^2+^ free conditions, the extracellular calcium is so low that its entry through 100 *μ*M NS3623-enhanced NSC is not enough to boost Gárdos channel activity.

This emphasizes the importance of calcium in red cell physiology as it reliably acts as a pervasive secondary messenger throughout the population, composed of billions of cells, quickly coordinating the overall response (Hertz et al., 2017). Lack of calcium compromises this and the response will depend more on the individual status of the cells such as ATP availability, protein oxidation, cell age (Baunbaek and Bennekou, 2008; Seear and Lew, 2011)… etc. *In vivo*, it may mean enabling an ultra-fast shrinking response for capillary passage together with Piezo1 mechanosensitive activity, as has been proposed (Dyrda et al., 2010; Danielczok et al., 2017b). Although calcium does not activate the cation conductance pathways it may directly either modulate it by affecting channel gating or open probability or, at the very least, favor a subsequent Na^+^ pathway opening. More interestingly, it may represent a fast feedback loop for transient hyperpolarization due to Gárdos channel activation in the narrowest of the capillaries. Indeed, it was shown that mechanical force causes calcium influx into RBCs that is dependent on Piezo1 expression (Cahalan et al., 2015). Such activation causes Ca^2+^ influx and eventually Gárdos channel-dependent RBC dehydration, with certainty the scenario leading to hereditary xerocytosis with many Piezo1 mutants described so far (Zarychanski et al., 2012; Cahalan et al., 2015; Andolfo et al., 2018; Rotordam et al., 2019; Peres et al., 2021). But for healthy RBCs, such transient activation (a few ms), will offer (i) a simple opportunity to adjust the cell volume without altering shape or deformability of the erythrocyte in capillaries below their own volume, (ii) back to the normal bloodstream, activation of a NSC will tend to let Na^+^ entering the cell, allowing near full restoration of cell volume, (iii) which eventually should give enough time for the Na^+^/K^+^ pump (considering the rate of exchange) to finely tune the intracellular Na^+^/K^+^ ratio to maintain homeostasis.

In conclusion, NSC activity can take place in hyperpolarizing conditions via pharmacological approach with a hitherto reported efficient chloride conductance inhibitor NS3623 and with calcium as a positive modulator. Except for Piezo1 agonist Yoda1 and Jedi-related compounds (Syeda et al., 2015, 2016), no activator of NSCs has been described. We argue that concomitant NSC enhancement and chloride inhibition is a key advantage, as it will allow studies of the cationic conductance's of the RBC with a single supplementary drug.

## Data Availability Statement

The original contributions presented in the study are included in the article/Supplementary Material, further inquiries can be directed to the corresponding author.

## Ethics Statement

The studies involving human participants were reviewed and approved by the institutional (CNRS) Ethical Committee and by the French Ministry for Research (declaration DC-2019-3842). The patients/participants provided their written informed consent to participate in this study, in accordance with the guidelines of the Helsinki Declaration of 1975, as revised in 2008.

## Author Contributions

DMA, LP, AH, GB, and SE defined the study, performed the experiments, interpreted the data, and drafted the manuscript. All authors listed have made a substantial, direct and intellectual contribution to the work, and approved it for publication.

## Funding

This study was supported by the European Framework Horizon 2020 under grant agreement number 675115 (RELEVANCE, DMA and SE), grant agreement number 860436 (EVIDENCE, AH and SE), and the Laboratory of Excellence GR-Ex, reference ANR-11-LABX-0051; GR-Ex is funded by the program Investissements d'avenir of the French National Research Agency, reference ANR-11-IDEX-0005-02 (SE, GB, and LP).

## Conflict of Interest

The authors declare that the research was conducted in the absence of any commercial or financial relationships that could be construed as a potential conflict of interest.

## Publisher's Note

All claims expressed in this article are solely those of the authors and do not necessarily represent those of their affiliated organizations, or those of the publisher, the editors and the reviewers. Any product that may be evaluated in this article, or claim that may be made by its manufacturer, is not guaranteed or endorsed by the publisher.
