# Supplementary material for: The Chloride Conductance Inhibitor NS3623 Enhances the Activity of a Non-selective Cation Channel in Hyperpolarizing Conditions
Source: Front Physiol. 2021 Oct 11;12:743094. doi: 10.3389/fphys.2021.743094 (PMC8543036; doi:10.3389/fphys.2021.743094)
Supplement: Supplementary file 1 [file Data_Sheet_1.PDF]

Supplementary Figure S1

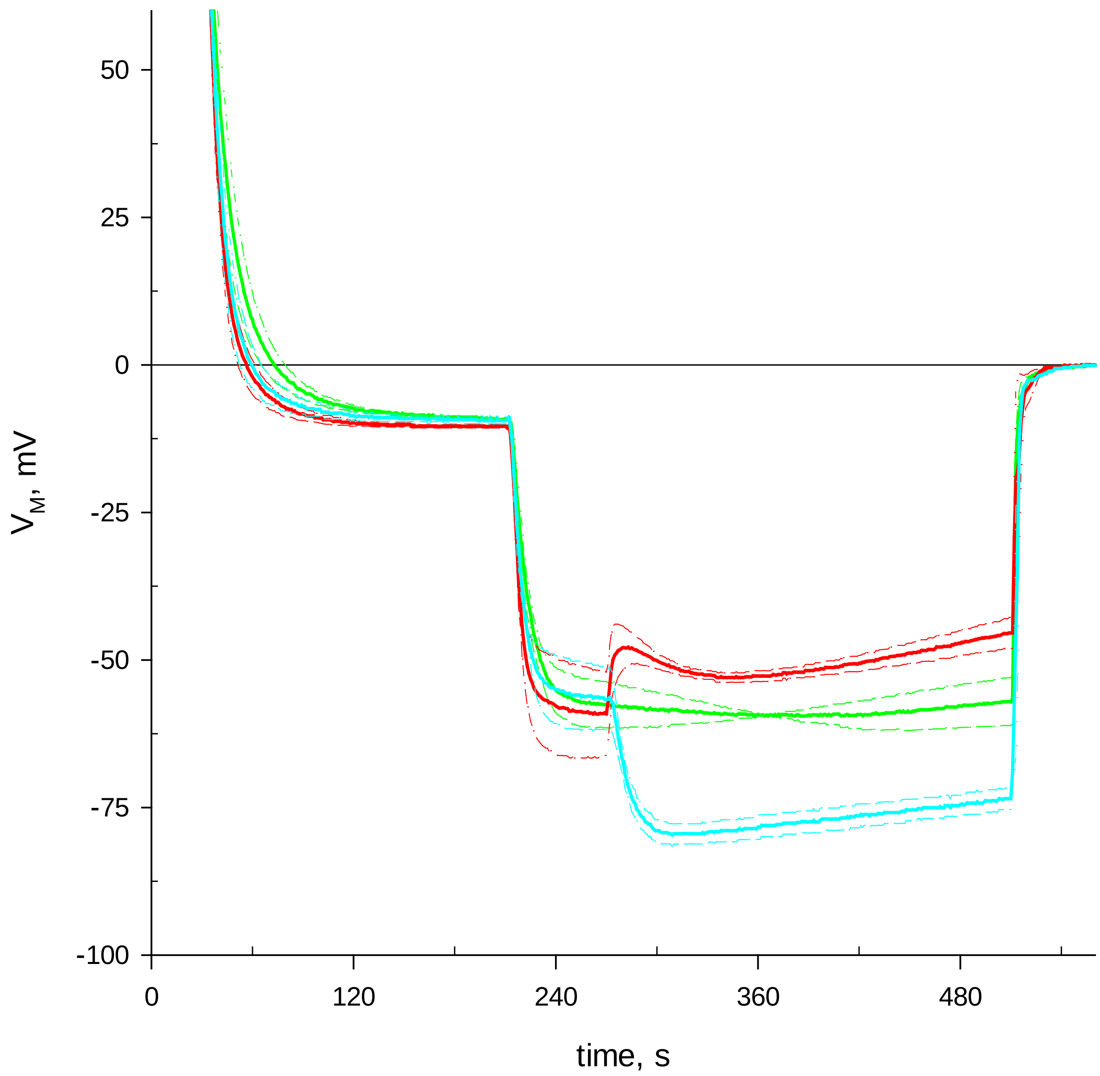

NS3623 inhibits the chloride conductance at 10 µM and becomes a potential NSC activator at 100 µM even when the hyperpolarization has already maximally developed.

Evolution of membrane potential from cells injected into normal Ringer solution at t=30 s with 0 µM (green, mid trace), 10 µM (cyan, bottom trace) or 100 µM (red, top trace) of chloride conductance inhibitor NS3623 provided after hyperpolarization was triggered by 10 µM A23187. Average of n=3 experiments, dashed lines indicate ± SEM.

Supplementary Figure S2

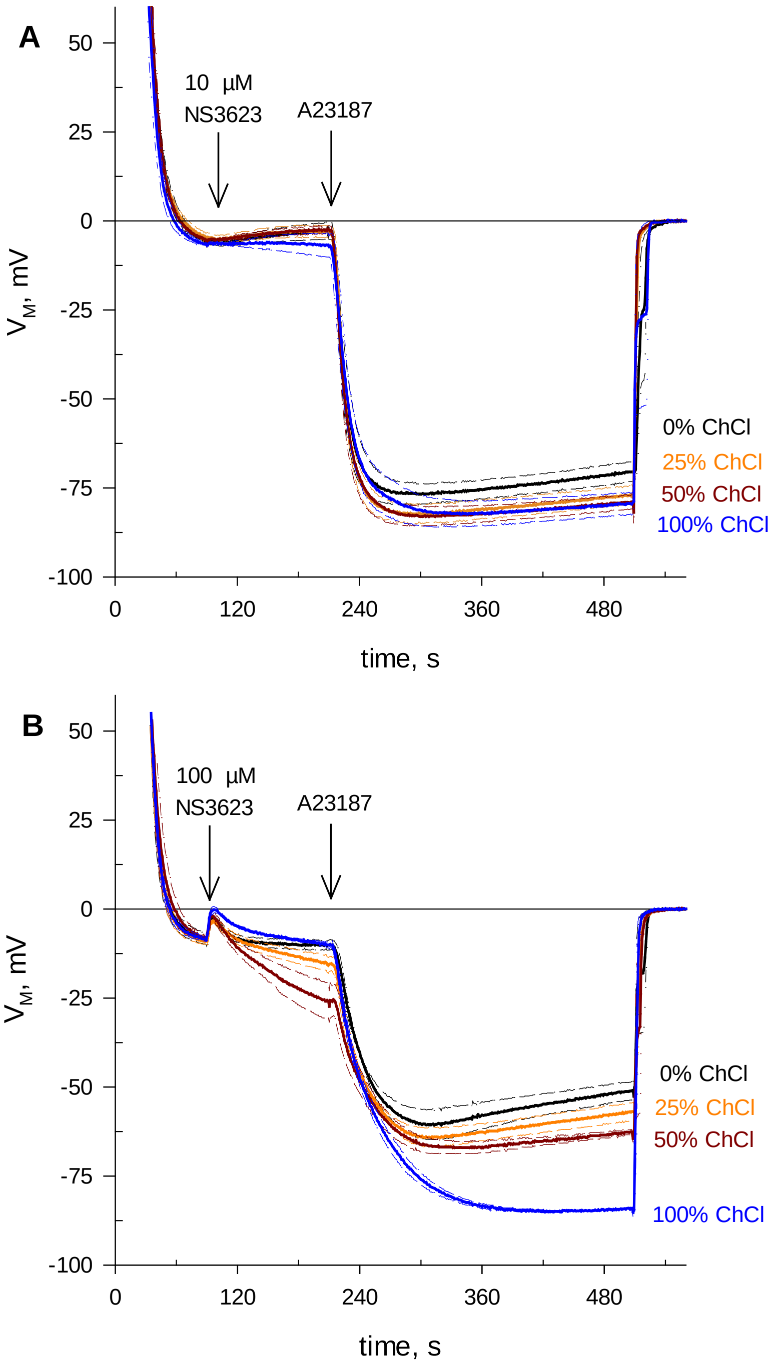

Removing Na^+ from the extracellular solution has barely no impact on Gárdos hyperpolarization after inhibiting the chloride conductance with 10 µM NS3623, whereas the use of 100 µM NS3623 clearly reveals a sodium dependency in the extent of hyperpolarization. This is further confirmed by the abolition of the repolarization occurring after A23187-induced hyperpolarization. It strongly suggests that 100 µM NS3623 enhances a NSC mediating Na^+ influx.

Evolution of membrane potential from cells injected into respective choline chloride-substituted Ringers, treated with 10 µM (**A**) or 100 µM NS3623 (**B**) and hyperpolarized with A23187 (10 µM). Average of n=3 experiments, dashed lines indicate ± SEM.

Supplementary Figure S3

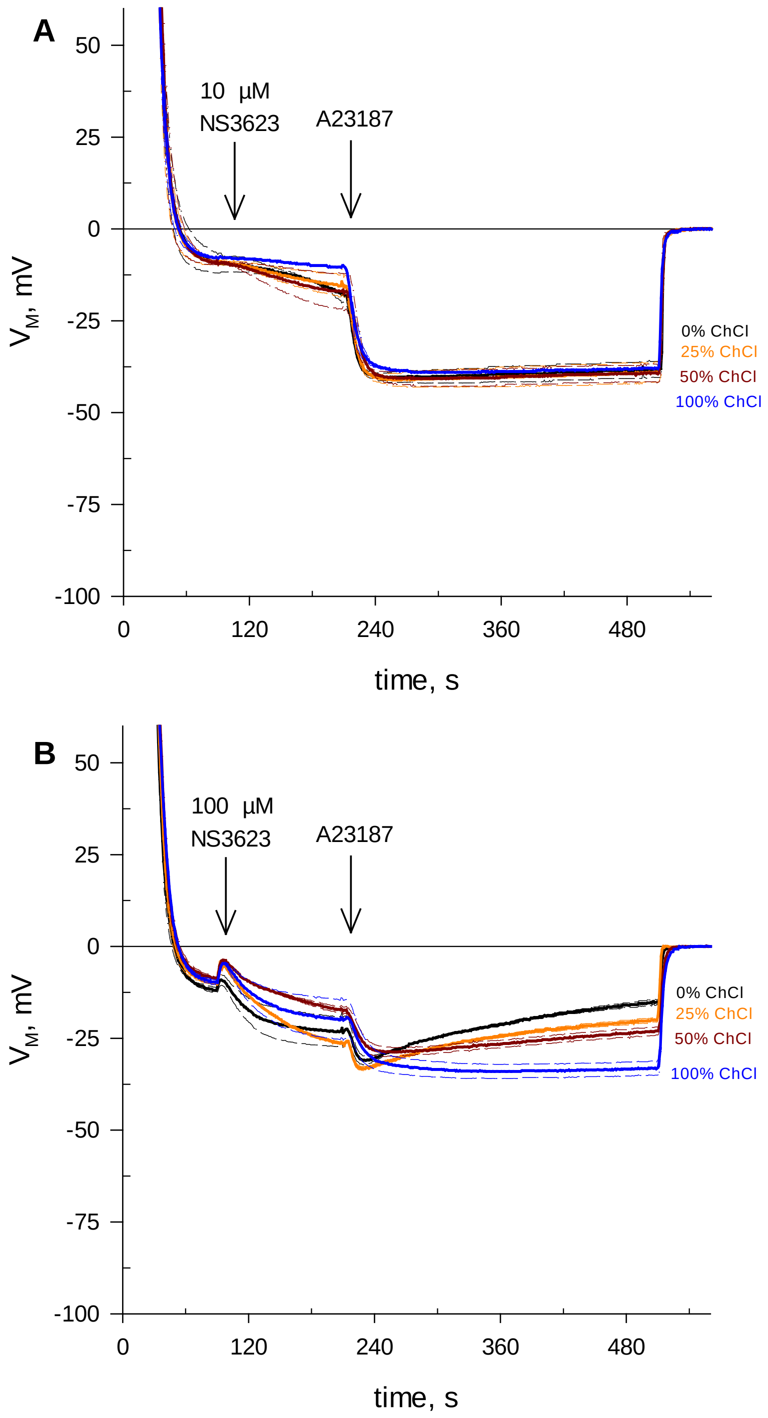

A smaller driving force for potassium via the use of a higher concentration (25 mM) in the extracellular solution does not preclude a differential hyperpolarization according to Na^+ available after use of 100 µM NS3623, pointing to enhanced NSC activity. NSC activity is absent or minimal at 10 µM NS3623.

Evolution of membrane potential from cells injected into respective choline chloride-substituted Ringers, treated with 10 µM (**A**) or 100 µM NS3623 (**B**) and hyperpolarized with A23187 (10 µM). Average of n=3 experiments, dashed lines indicate ± SEM. Traces/solutions, top to bottom: 0, 25, 50 and 100 % Choline Chloride (ChCl) degree of substitution, with 0% = 131 mM NaCl; 25 mM KCl and 100% 131 mM ChCl; 25 mM KCl.
