# Supplementary material for: The Chloride Conductance Inhibitor NS3623 Enhances the Activity of a Non-selective Cation Channel in Hyperpolarizing Conditions
Source: Front Physiol. 2021 Oct 11;12:743094. doi: 10.3389/fphys.2021.743094 (PMC8543036; doi:10.3389/fphys.2021.743094)
Supplement: Supplementary file 2 [file Data_Sheet_2.PDF]

Hyperpolarizing agent	Solution	NS3623 (µM) 0	10	100
A23187 (10µM)	Normal Ringer	-59.9±1.6 (22)	-85.3±0.8 (12)	-60.6±1.5 (4)
	100% Choline Ringer	n.d	-81.7±1.9 (3)	-86.1±0.8 (3)
NS309 (100 µM)	Normal Ringer	-26.5±6.3 (3)	-68.7±3.0 (3)	-31.5±5.1 (3)
		-0.21±0.07 (3)	-0.79±0.04 (3)	-0.09±0.03 (3)
	Calcium Ringer	-44.1±1.4 (3)	-87.3±1.4 (3)	-81.2±1.2 (3)
		-0.44±0.06 (3)	-1.29±0.09 (3)	-0.35±0.05 (3)

Supplementary Table 1. Summary of mean values of maximum hyperpolarization (mV, black) and initial rate of hyperpolarization (mV/s, red) obtained in this study upon the different conditions used with different concentrations of NS3623.
